# An integrated flow cytometry-based platform for isolation and molecular characterization of circulating tumor single cells and clusters

**DOI:** 10.1038/s41598-018-23217-5

**Published:** 2018-03-22

**Authors:** Neha Bhagwat, Keely Dulmage, Charles H. Pletcher, Ling Wang, William DeMuth, Moen Sen, David Balli, Stephanie S. Yee, Silin Sa, Frances Tong, Liping Yu, Jonni S. Moore, Ben Z. Stanger, Eric P. Dixon, Erica L. Carpenter

**Affiliations:** 1 Division of Gastroenterology, Department of Medicine, Perelman School of Medicine, University of Pennsylvania, Philadelphia, PA USA; 2 BD Technologies and Innovation, Research Triangle Park, NC, USA; 3 Department of Pathology and Laboratory Medicine, Perelman School of Medicine, University of Pennsylvania, Philadelphia, PA USA; 4 Division of Hematology and Oncology, Department of Medicine, Perelman School of Medicine, University of Pennsylvania, Philadelphia, PA USA; 5 BD Biosciences, San Jose, CA USA

**Keywords:** Biotechnology, Tumour biomarkers

## Abstract

Comprehensive molecular analysis of rare circulating tumor cells (CTCs) and cell clusters is often hampered by low throughput and purity, as well as cell loss. To address this, we developed a fully integrated platform for flow cytometry-based isolation of CTCs and clusters from blood that can be combined with whole transcriptome analysis or targeted RNA transcript quantification. Downstream molecular signature can be linked to cell phenotype through index sorting. This newly developed platform utilizes in-line magnetic particle-based leukocyte depletion, and acoustic cell focusing and washing to achieve >98% reduction of blood cells and non-cellular debris, along with >1.5 log-fold enrichment of spiked tumor cells. We could also detect 1 spiked-in tumor cell in 1 million WBCs in 4/7 replicates. Importantly, the use of a large 200μm nozzle and low sheath pressure (3.5 psi) minimized shear forces, thereby maintaining cell viability and integrity while allowing for simultaneous recovery of single cells and clusters from blood. As proof of principle, we isolated and transcriptionally characterized 63 single CTCs from a genetically engineered pancreatic cancer mouse model (n = 12 mice) and, using index sorting, were able to identify distinct epithelial and mesenchymal sub-populations based on linked single cell protein and gene expression.

## Introduction

Circulating tumor cells (CTCs) are rare cells shed from solid tumors and found at extremely low numbers in the bloodstream of patients in most cancer types. A subset of these cells can seed distant organs in the body and give rise to metastases, which are the primary cause of cancer-related mortality^1^. Sampling these cells in the form of a ‘liquid biopsy’ can be a sensitive and non-invasive method for early detection, disease monitoring, and identification of therapeutic targets. Indeed, a number of studies have demonstrated the clinical utility of this approach; CTC number is correlated with a worse prognosis in most carcinomas^2–6^ and CTC analysis has been used to detect actionable mutations or the development of acquired resistance to targeted therapies^7–9^. Transcriptional characterization of CTCs at a single-cell level can provide additional insights into tumor heterogeneity, and identify clinically relevant signaling pathways for therapeutic intervention^10–12^.

In addition to individual CTCs, there is emerging evidence demonstrating the clinical importance of circulating tumor cell clusters in blood^13–16^. The presence of CTC clusters is associated with increased metastatic potential^17,18^ and lower progression free survival in breast, prostate and lung cancer^19–22^. However, the extremely low frequency of occurrence of both single cells and clusters in the blood (~1–100 cells in a background of billions of blood cells) makes isolation and detailed analysis of these cells challenging.

CellSearch® is the only FDA-approved platform for clinical characterization of CTCs. However, this approach offers only enumeration and limited phenotypic analysis with just one open channel for the addition of new markers. It also does not yield purified viable cells that can easily be used for downstream molecular analysis or functional studies. The end product is an enriched fraction of CTCs that may also include clusters^20,23^, although the CellSearch system was not specifically designed to capture CTC clusters. Size-based enrichment^15,16,24,25^ can miss the fraction of CTCs that are equal to or smaller than WBCs^26,27^. In recent years, a number of groups have developed methodologies for bulk CTC enrichment based on immunocapture of surface proteins^28–31^, negative depletion of hematopoietic cells^32,33^, and direct imaging^34^. For single-cell analysis, the enriched CTCs often have to go through an additional purification step such as the DEPArray^27,35^, Fluidigm C1^36^ or single-cell micro-manipulation^37^. However, this leads to additional loss during transfer^35^ and these approaches can be time- and labor-intensive, and thus less compatible with deployment in a clinical lab setting.

While it has prognostic value, CTC count alone is rarely clinically actionable. Tumor molecular subtyping based on transcriptional profiles^38,39^ and detection of targetable variants^40^ are increasingly relevant for therapy selection in pancreatic and other cancers. However, repeat access to tissue samples can be difficult or impossible^41,42^, suggesting a role for CTC-based molecular monitoring. Therefore, to be clinically relevant, it is critical to have an integrated next-generation CTC analysis platform that is capable of (i) efficiently isolating single cells as well as clusters at the same time, (ii) providing pure cell populations with minimal or no WBC contamination, and (iii) high-throughput retrieval of viable cells for molecular analysis. Additionally, the platform must be readily adaptable for multiplex positive- or negative-selection approaches for multiple cancers with diverse cell surface protein markers, and have single-use tubing kits available for eventual use for clinical tests. To our knowledge, none of the existing platforms fulfill all the above criteria.

In this report, we describe a novel flow cytometric approach that integrates isolation of rare circulating tumor single cells and clusters from whole blood with whole transcriptome analysis (WTA) and a novel BD Precise™ technology^43^ for accurate quantification of RNA transcripts in single cells in a low-cost and high-throughput format. This method combines immunomagnetic depletion of leukocytes and red blood cells (RBCs) followed by acoustic cell washing and focusing to pre-enrich tumor cells in the blood prior to cell sorting. Additionally, we utilized flow cytometric index sorting^44^ that allows for the correlation of phenotypic and molecular profiles at the single cell level. Using this workflow, we were able to successfully isolate rare CTCs from a genetically engineered autochthonous mouse model of pancreatic ductal adenocarcinoma (PDA) and identify distinct epithelial and mesenchymal sub-populations based on protein and transcriptional signatures. Thus, we demonstrate an integrated, clinically feasible workflow for isolation and molecular characterization of CTCs.

## Results

### Approach to rare cell pre-enrichment, isolation, and analysis

**Figure 1:**
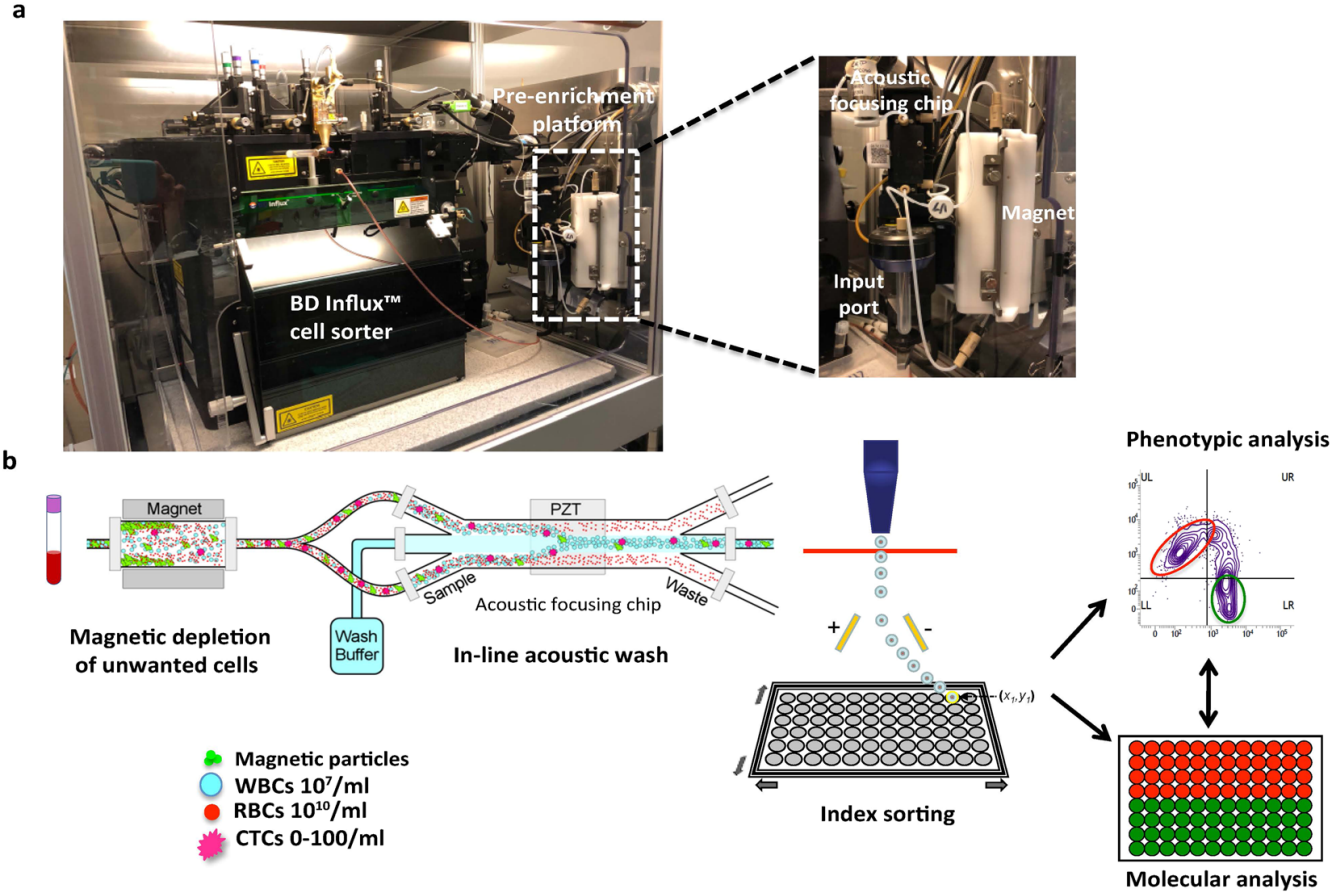
Schematic of integrated platform and workflow. (**a**) Pre-enrichment platform is connected in-line with the BD Influx™ cell sorter. (**b**) Whole blood is labeled with antibodies against CTC markers as well as magnetic microparticles that bind unwanted blood cells. The sample then passes through a magnetic depletion step that removes >98% of unwanted blood cells followed by an in-line acoustic focusing and washing step, which removes debris and concentrates the sample prior to cell sorting. The sample can be interrogated based on cell markers and single cell or bulk populations of interest can be easily index-sorted for correlation of flow phenotype with molecular profile. WBC – White blood cell, RBC – Red blood cell, CTC – Circulating tumor cell, PZT – lead zirconate titanate.

We developed an integrated magnetic separator and acoustic microfluidic platform^45^ for rare cell pre-enrichment from whole blood. This pre-enrichment platform is connected in-line with the BD Influx^TM^ cell sorter and has a small footprint compared to the flow sorter to which it is attached (Fig. 1a). The sample input port, magnet, acoustic focusing chip, camera, and flow sensors are grouped close together to minimize dead volumes and maximize sensitivity. The magnetic separator is fully enclosed and uses low-cost disposables to maintain sample sterility.

Whole blood is prepared for cell sorting by labeling with fluorescently conjugated antibodies, along with BD IMag™ magnetic particles that can be covalently conjugated to monoclonal antibodies or streptavidin to deplete unwanted blood cells. This is followed by the addition of a gentle RBC lysis buffer that preserves CTC viability. The sample is then directly run through the instrument, without additional centrifugation-based wash steps, which could lead to cell loss. It first passes through tubing enclosed by a magnetic separator that depletes unwanted leukocytes and RBCs that have been magnetically labeled. The sample then flows through an acoustic focusing chip in which ultrasonic standing waves are used to separate particles based on their size, density and compressibility^46,47^. Based on this principle, smaller particles such as cell debris, unbound antibodies, RBCs, and platelets will be displaced further from the central channel as compared to larger nucleated cells including CTCs, which are directed to the cell sorter. At the same time, the sample can be washed without centrifugation by flowing wash buffer through the central channel during acoustic focusing, thereby further minimizing sample manipulation and cell loss associated with centrifugation^48^. The density of the wash buffer can be varied to achieve optimum cell separation. Detailed and quantitative phenotypic analysis can be performed on individual cells by flow cytometry, which can then be recovered through sorting for downstream molecular analysis. Further, the index-sorting feature, in which a high content cytometric phenotype of every individual sorted cell is recorded, allows for retrospective analysis of sorted single cells based on their molecular profile (Fig. 1b).

### Platform performance characteristics

**Figure 2:**
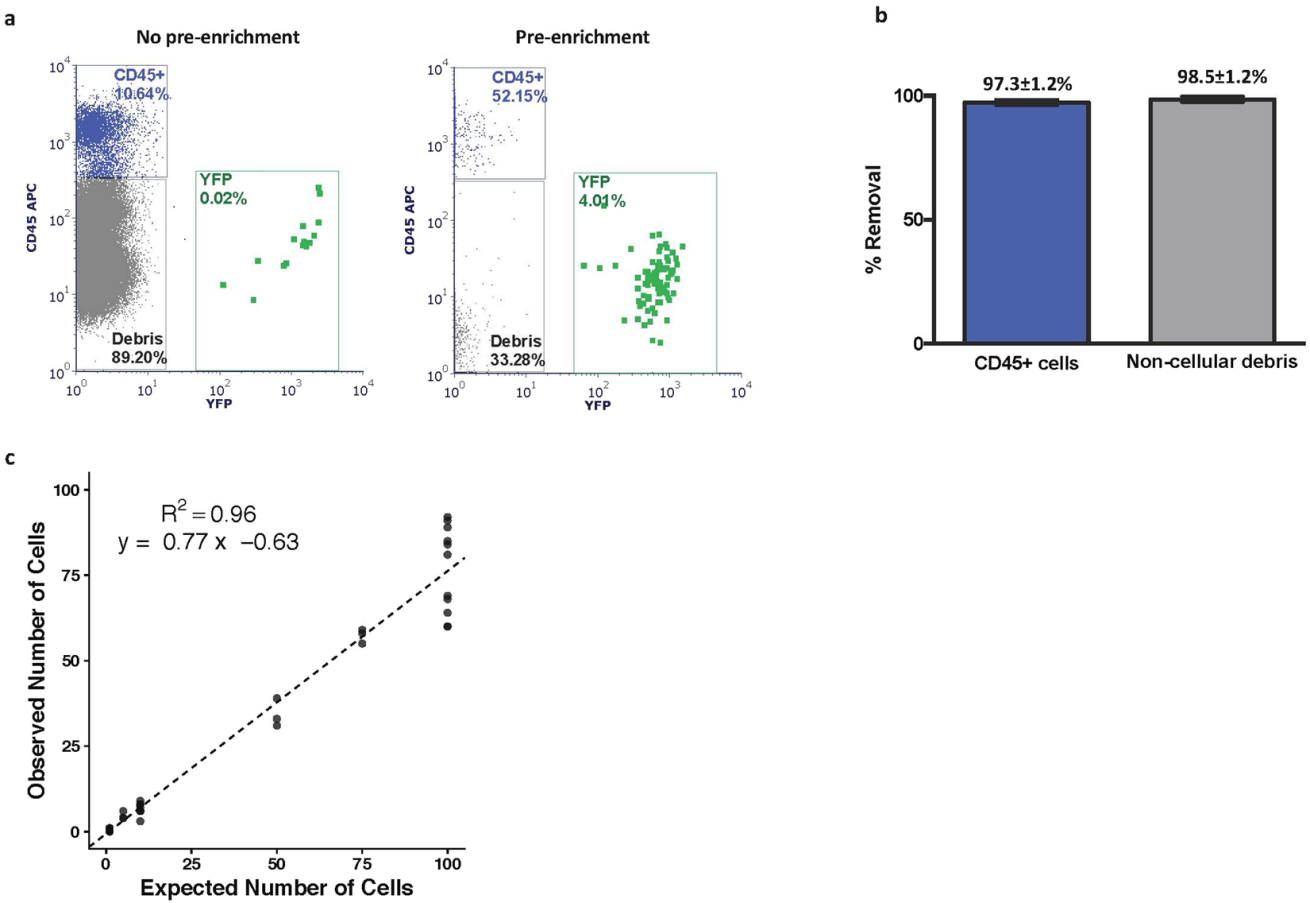
Performance characteristics of platform. (**a**) YFP+ PD7591 cell line cells were spiked into normal mouse blood and processed through BD Influx™ only (no pre-enrichment; left) or together with the pre-enrichment workflow (with pre-enrichment; right). (**b**) Percent removal of CD45+ WBCs and non-cellular debris with pre-enrichment platform as compared to without pre-enrichment (n = 5, mean ± SD). (**c**) Average calculated cell recovery using linear regression analysis (n = 3–9).

To optimize and measure the platform performance characteristics, we first utilized a tumor-derived cell line (PD7591) from a genetically engineered mouse model of pancreatic cancer (*Pdx1-Cre*, *Kras*^*LSL-G12D*^*, p53*^*L*/+^, *Rosa*^*YFP*/*YFP*^ (KPCY))^49^. Cultured PD7591 cells expressing yellow fluorescent protein (YFP), that could be detected by flow cytometry in a marker-independent manner, were spiked into healthy mouse blood. The sample was stained with CD45-APC for WBCs and DAPI as a viability marker, and also labeled with magnetic particles targeting APC (to deplete WBCs) and Ter-119 (to deplete RBCs). The sample was then diluted with RBC lysis buffer to achieve a gentle lysis of RBCs. To determine efficiency of enrichment, half the unwashed sample was run through the pre-enrichment platform in-line with the BD Influx™, and the other half of the sample was run directly on the Influx, without pre-enrichment. YFP and CD45 positive cells were assessed by flow cytometry, with representative results shown in Fig. 2a. The sample processing rate was 6 ml/hr of diluted blood. We were able to achieve 97.3 ± 1.2% reduction in white blood cells and 98.5 ± 1.2% reduction in debris with the pre-enrichment platform (Fig. 2b). This resulted in a 95% decrease in event rate on the flow cytometer and >1.5 log enrichment in target cell population.

To assess post-enrichment cell recovery, 1, 5, 10, 50, 75 and 100 YFP+ cells were directly sorted into whole blood and each sample was labeled, lysed, pre-enriched, and cell recovery analyzed by flow cytometry. The observed cell counts were plotted against expected cell counts (Fig. 2c). A linear regression analysis was performed, and the average recovery was calculated to be 77% (R^2^ = 0.96). We examined possible sources of cell loss and found that approximately 10% of spiked-in cells were found in the waste from the acoustic focusing chip, an additional 5% of cells were bound in the magnetic tubing, and the remaining fraction was likely lost in the dead volume of the tubing. We were able to reliably detect 1 cell in 1 million WBCs (as assessed by a hemocytometer) in 4 out of 7 replicates. Thus, we demonstrate high cell recovery and sensitivity using the integrated pre-enrichment platform and Influx cell sorter.

### Minimal effect of processing on molecular profile, phenotype, and viability

**Figure 3:**
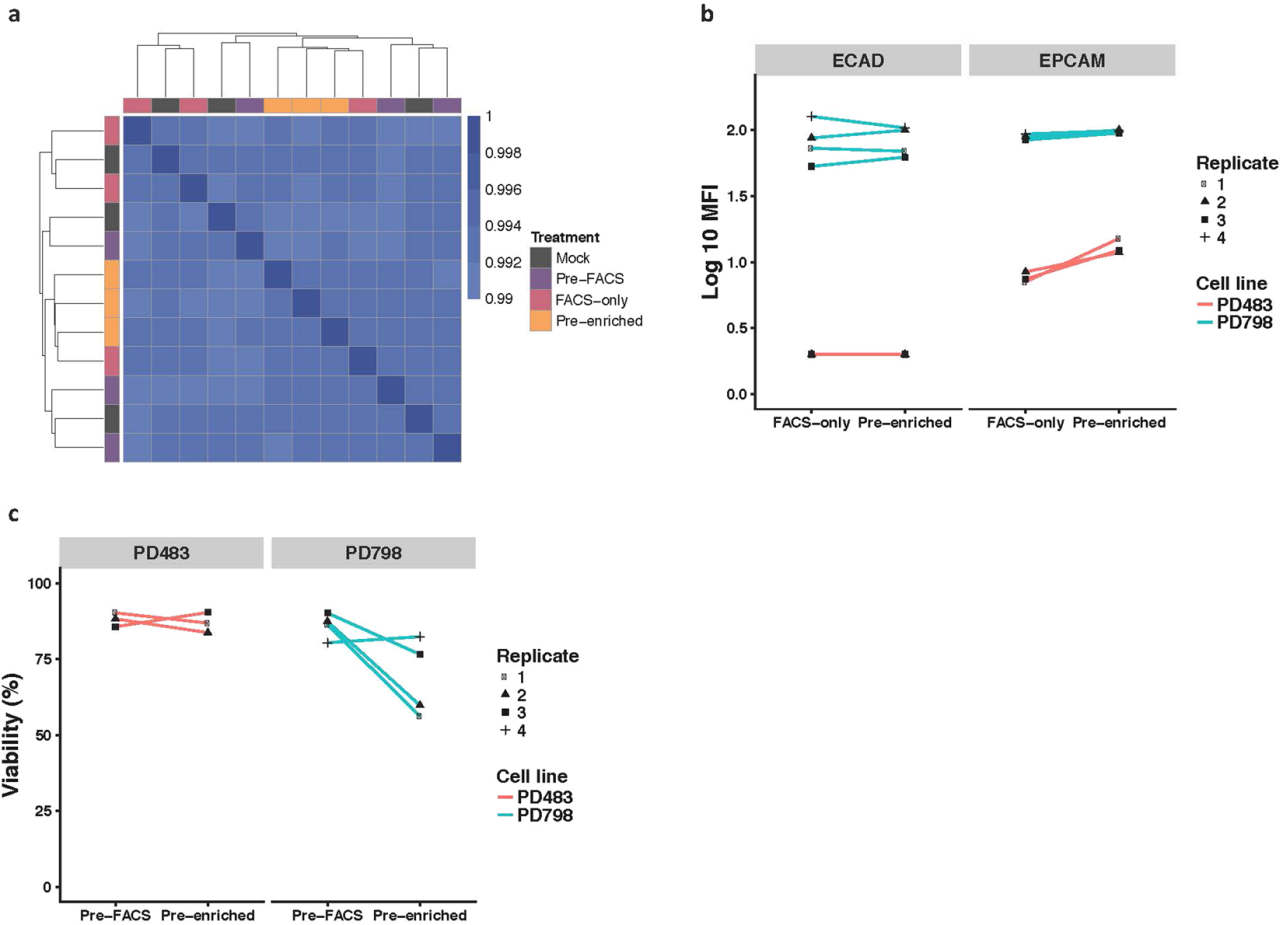
Sample processing has minimal effects on gene expression, cell phenotype and viability. (**a**) Correlation matrix comparing gene expression profiles as determined by whole transcriptome RNA sequencing. PD798 cells were sorted through the pre-enrichment workflow (Pre-enriched), sorted through Influx only (FACS-only), analyzed prior to sorting (Pre-FACS), or were incubated in buffer only on the bench (Mock). Scale refers to Pearson correlation coefficient. (n = 3). 13845 genes were considered in the analysis. (**b**) The PD483 and PD798 cell lines were processed by FACS-only or with pre-enrichment to determine the processing effects on cell surface protein expression of EPCAM and ECAD. (MFI – mean fluorescence intensity) (**c**) Viability was assessed by Trypan Blue exclusion in the same two cell lines, PD483 and PD798, and was not significantly different at the end of the pre-enrichment workflow as compared to before sorting. Wilcoxon paired signed-rank test as well as a linear mixed-effects model was performed to determine statistical differences.

Given our intention of sorting rare cells for molecular analysis, and linking molecular profile to phenotype using index sorting, we next sought to measure any changes in gene expression, cell viability, or phenotype induced by processing. To measure changes in gene expression, the KPCY tumor-derived cell line PD798 was incubated with magnetic particles, treated with RBC lysis buffer, and sorted using the pre-enrichment workflow as described above (referred to as “pre-enriched” in Fig. 3a). As a comparison, the same sample was also sorted through the BD Influx™, without passing through the pre-enrichment step (“FACS-only”). An aliquot of PD798 was also banked prior to sorting as a pre-sort control (“pre-FACS”). Finally, an identical sample was incubated in staining buffer at room temperature for the same time period, referred to as “Mock.” RNA was extracted from bulk cells from all samples, and whole transcriptome RNA sequencing was conducted to assess potential effects on gene expression. The transcriptional profiles across all 4 conditions were highly similar (R > 0.99), and replicate analysis from any one condition demonstrated that within-condition expression profiles were not more highly correlated than between-condition expression profiles when unsupervised hierarchical clustering was performed (Fig. 3a). Similar results were also observed using a second KPCY-derived cell line, PD483 (Supplementary Fig. S1). We also performed differential gene expression analysis between all processing conditions and found 2 out of 13,845 genes in PD798 (0.01%), and 7 out of 13,264 genes in PD483 (0.05%) to be significantly different between the various conditions. Taken together, these results demonstrate that the pre-enrichment workflow does not significantly affect gene expression.

We next sought to determine whether exposure to the RBC lysis buffer followed by on-chip washing had an effect on the detection of the cell surface proteins EPCAM and E-cadherin (ECAD) by flow cytometry. Using the KPCY tumor-derived cell lines PD798 (epithelial characteristics with high ECAD expression) and PD483 (mesenchymal characteristics with low ECAD and EPCAM expression) (Supplementary Fig. S2), and comparing cells processed by FACS-only to those that were also pre-enriched, no difference in the mean fluorescence intensity of the cell-surface proteins could be detected (Fig. 3b). Cell viability as measured by Trypan Blue exclusion was not found to be significantly different at the end of cell sorting as compared to unmanipulated cells prior to sorting (Fig. 3c). Taken together, these results indicate that the assay workflow including RBC lysis is compatible with phenotypic analysis as well as RNA-based molecular profiling.

### Isolation of cell clusters

**Figure 4:**
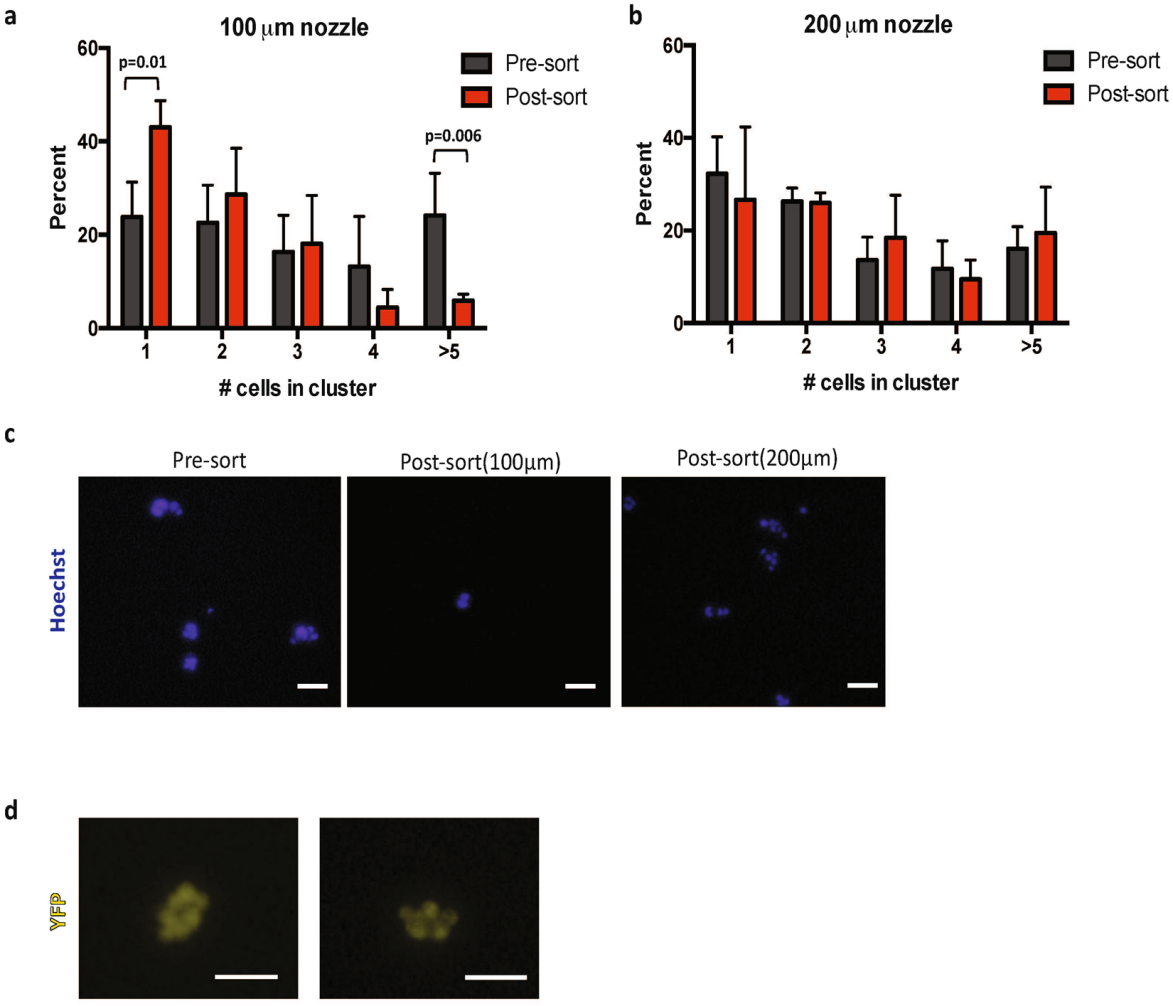
Optimized workflow preserves integrity of cell clusters. (**a**) Distribution of cell clusters before and after being sorted through (**a**) standard 100 μm nozzle and (**b**) 200 μm nozzle, and following pre-enrichment. Use of 100 μm nozzle results in a significant decrease in frequency of large clusters (>5 cells) and a corresponding increase in number of single cells whereas use of 200 μm nozzle maintains integrity of cell clusters. A negative binomial generalized linear model was used to determine statistical significance (n = 4) (**c**) Representative images of cells from (**a**) and (**b**). (**d**) Circulating cell clusters recovered from blood of a tumor-bearing KPCY mouse in which pancreatic tumor cells are labeled with YFP. Scale bar: 100 μm.

Recent studies suggest CTC clusters may have increased metastatic potential and higher prognostic value as compared to single cells^14,17^, and yet most approaches for rare cell purification are capable of isolating either single cells or clusters, but not both at the same time^24,32^. To optimize our approach for simultaneous isolation of single cells and clusters, we utilized a 200 μm nozzle on the Influx, which is twice as large as standard nozzles used in cell sorters. In comparison to the standard 100 μm nozzle, this led to a > 5-fold decrease in sheath pressure from 20 psi to 3.5 psi (180 mmHg), which would be predicted to preserve cell clusters. In order to assess the effect of the workflow on the integrity of cell clusters, we spiked *in vitro* generated cell clusters^17^ into whole mouse blood, incubated the sample with antibodies and magnetic beads and sorted using the above described pre-enrichment workflow. Cluster size (number of cells present), as well as total number of clusters was measured by microscopy prior to sorting and at the end of the sort. The experiment was repeated using a standard 100 μm nozzle as a control. The use of a smaller nozzle resulted in a significant decrease in the number of larger cell clusters (>5 cells) and a corresponding increase in single cells after sorting, indicating that large cell clusters were dissociating into individual cells under these conditions (Fig. 4a). However, the distribution of cluster size was maintained with the use of the larger 200 μm nozzle, suggesting that cell clusters in the blood remain intact under the low shear stress exerted under these conditions (Fig. 4b). Representative images from both conditions are shown in Fig. 4c. Using the same workflow, we were also able to successfully isolate circulating tumor cell clusters of >5 cells from whole blood obtained from the KPCY mouse model of pancreatic cancer in which all tumor cells express YFP (Fig. 4d). Thus, the gentle sample processing and minimal manipulation associated with this workflow allows for the isolation and recovery of intact cell clusters as well as single cells.

### RNA Sequencing of single and pooled cells

**Figure 5:**
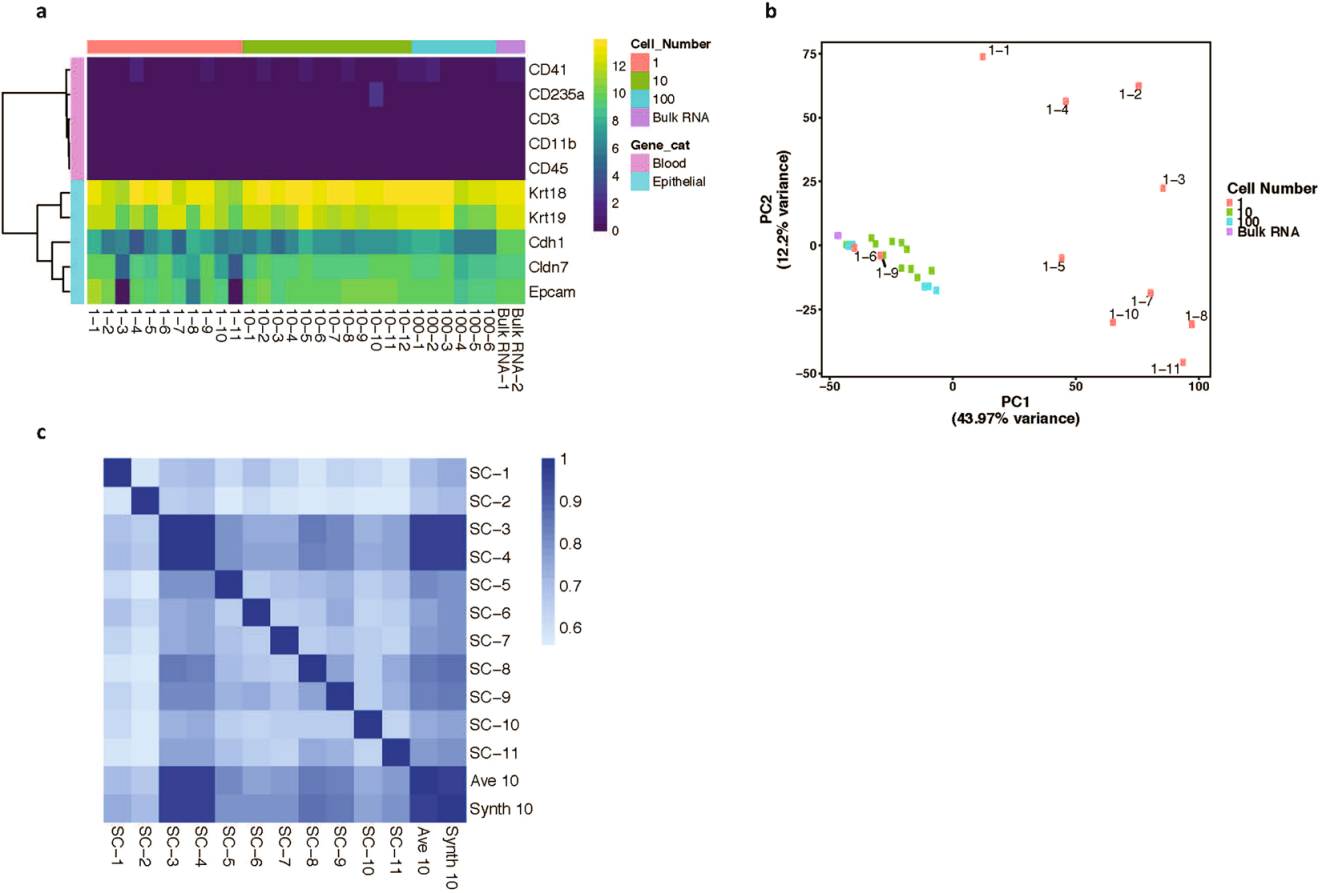
Whole transcriptome analysis of sorted single and pooled cells. (**a**) RNA sequencing was conducted on single cells (n = 11), 10-cell (n = 12) and 100-cell (N = 6) pools, and bulk RNA (n = 2) from PD798 cells pre-enriched and sorted from whole blood. Expression of selected hematopoietic lineage genes is extremely low or undetectable in all sorted cell populations, indicating high purity. Scale refers to log 2 TPM (transcripts per million). (**b**) Principal component analysis of gene expression of the 1,000 most variable genes in the different sorted cell populations or bulk RNA. (**c**) Correlation matrix comparing gene expression profile (13,817 genes) of single cells to the sum of all profiles (Synth 10) and the average of 10 cell pools (Ave 10). Scale refers to Pearson correlation coefficient. SC: Single cell.

Detailed molecular characterization of CTCs, particularly at the gene expression level, can be invaluable for assessing tumor molecular heterogeneity and potentially informing clinical management. Along with efficient detection and isolation of rare CTCs, we wanted to ensure that our workflow could be easily integrated with complex downstream analysis including next-generation sequencing. Further, we wanted to assess the purity of the recovered cells, which is critical for performing meaningful single-cell analysis. PD798 cells were spiked into healthy mouse blood and enriched as described above. Single cells (n = 11) as well as pools of 10 (n = 12) and 100 (n = 6) cells were sorted and cDNA libraries were prepared with cell lysate as input using the Clontech SMART-Seq® v4 kit. RNA isolated from the bulk cell line (n = 2) was used as a control. We were able to successfully generate high-quality libraries from 11/13 single cells (85% success rate) and from 18/18 pools of cells. The samples were sequenced at a mean depth of 5.8–15.3 million reads, of which a mean of 86.68% uniquely aligned to the mouse reference genome (Supplementary Fig. S3) The recovered cells had minimal WBC contamination based on very low expression of hematopoietic lineage markers in the sorted cell populations, in single cells as well as for sorted pools (Fig. 5a). Principal component analysis of the gene expression showed that sample inputs of 10 or more cells cluster together with the bulk RNA while heterogeneity is seen within the single cell population (Fig. 5b). However, the sum of 10 single cell profiles (“Synth 10”) was highly correlated with the expression of 10 cell bulk samples (“Ave 10”), suggesting that single cell heterogeneity observed in this experiment is normally masked when bulk samples are sequenced (Fig. 5c). This workflow, therefore, is well suited for performing detailed molecular analysis including whole transcriptome sequencing on highly pure populations of rare cells.

### Targeted gene expression profiling of single cells

**Figure 6:**
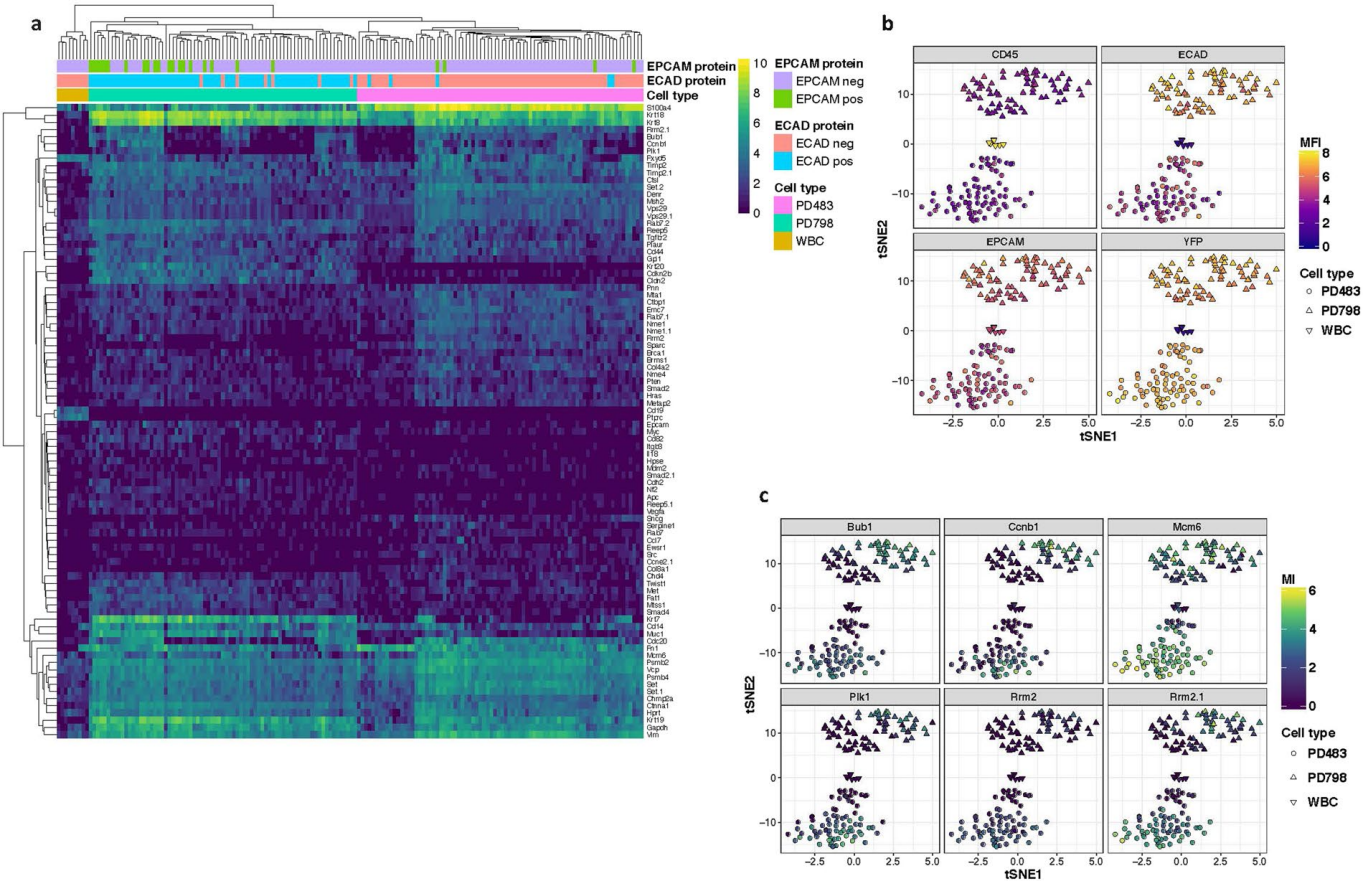
Integration of single cell sorting with BD Precise™ assay. (**a**) Heat map showing unsupervised hierarchical clustering of Molecular Index (MI) counts of 88 transcripts representing 78 genes in PD798, PD483, and WBCs. For visualization purposes, genes with total counts lower than 64 MI (1 SD below mean) were filtered out. Scale bar refers to log 2 MI counts. Expression of EPCAM and ECAD protein, as detected by flow cytometry, is denoted above heat map. (**b**) t-SNE analysis of gene expression of PD798 (triangle), PD483 (circle) and WBCs (inverted triangle) colored by log 10 mean fluorescence intensity (MFI) of CD45 (upper left), ECAD (upper right), EPCAM (lower left), and YFP (lower right). (**c**) t-SNE plots colored by expression of cell cycle genes *Bub1, Ccnb1, Mcm6, Rrm2, Rrm2.1* (designates alternate *Rrm2* transcript), and *Plk1* (starting with upper left and moving clockwise). Scale bar refers to log 2 MI counts.

Whole transcriptome sequencing (WTS) is essential for discovery of molecular signatures associated with various states of disease and responsiveness to therapy. However, the expense and low throughput nature of WTS makes its use as a clinical diagnostic impractical. As a lower-cost alternative for the analysis of already identified correlative biomarkers, we integrated the pre-enrichment workflow with BD Precise™ assays, which offer highly sensitive and unbiased quantitation of a targeted gene panel for single or pooled cells. Additionally, the single-cell molecular data was correlated with phenotypic characteristics using the index-sorting function. PD798 and PD483 cell lines were each spiked into healthy mouse blood and stained with antibodies against epithelial markers EPCAM and ECAD along with the leukocyte marker CD45. The sample was then labeled with magnetic microparticles to deplete CD45+ WBCs and Ter-119+ RBCs, followed by RBC lysis. After passing through the pre-enrichment platform, individual CD45- YFP+ cells were sorted into a 96-well Precise™ encoding plate containing cell lysis buffer, molecule-specific barcodes (Molecular Index), and sample barcodes. 10-cell pools of CD45+ WBCs were also sorted as controls. Cells were sequenced using a custom panel comprising 152 genes involved in metastasis and epithelial to mesenchymal transition (EMT) (Supplementary Table S1). We were able to successfully sequence >97% of the sorted single cells. Unsupervised hierarchical clustering of gene expression measured by molecular counts demonstrated a clear distinction between the two different cell lines and WBCs (Fig. 6a). The gene expression profiles of WBCs sorted from the two different experiments clustered together, indicating that the assay is reproducible. PD483 had high expression of mesenchymal markers including *S100a4, Sparc* and *Col8a1* and low expression of epithelial markers such as *Ecad* (*Cdh1*)*, Epcam, Krt7, Krt19* and *Muc1* whereas expression in PD798 was the opposite. Expression of hematopoietic genes like *Ptprc* and *Cd19* was low or negative in sorted tumor cells compared to WBCs, again indicating high purity (Supplementary Fig. S4). T-distributed stochastic neighbor embedding (t-SNE), the preferred method for dimensional reduction of single cell expression data, was able to recapitulate three distinct molecular profiles, corresponding to the two different cell lines and WBCs. Further, since the cells were index-sorted, the flow phenotype i.e. fluorescence intensity of YFP, CD45, EPCAM and ECAD were superimposed on the molecular profile. As expected, the tumor cells were strongly positive for YFP and negative for CD45 staining. Also, PD798 had higher expression of surface ECAD and EPCAM as compared to WBCs and PD483 (Fig. 6b). Further, we observed distinct sub-populations within the two cell lines, which correlated with expression of cell cycle genes (Fig. 6c), suggesting that some of the heterogeneity within each cell line might be due to differences in cell cycle status.

### Single cell analysis of index-sorted CTCs from a pre-clinical model of pancreatic cancer

We applied the entire integrated workflow to sort and perform molecular analysis of rare CTCs from a preclinical model of pancreatic cancer (*Pdx1-Cre*, *Kras*^*LSL-G12D*^*, p53*^*R172H*/+^)^50^ with a yellow fluorescent protein (YFP) lineage tag (*Rosa*^*YFP*/*YFP*)^), in which all pancreatic epithelial cells express YFP. We have previously shown in a similar model that YFP+ cells of pancreatic lineage can be detected in the circulation of these mice and can seed distant sites even at early stages of disease progression^49^. As proof of principle, whole blood was stained with antibodies against EPCAM, ECAD, and CD45 and processed through the pre-enrichment workflow. From the enriched fraction, we then sorted 477 YFP +CTCs from 12 tumor-bearing mice with high metastatic burden (Mean = 47 cells/ml, range 1–254 cells/ml) as individual cells (n = 157) or into 10-cell pools (n = 32). Single cell and 10-cell pools of YFP- CD45+ WBCs were also sorted from each sample as a control. Additionally, matched tumor from each mouse was dissociated, and 650 total single cells were index-sorted for comparison. Using the BD Precise™ workflow, we were able to successfully sequence 40% of single CTCs and 73.8% of single tumor cells. We were unable to generate high-quality libraries from single WBCs (>90% failure rate), therefore 10-cell pools were used for analysis. In total, we obtained sequencing data from 63 individual CTCs, 30 ten-cell pools of CTCs, 480 single tumor cells, and 26 ten-cell pools of WBCs for the final analysis.

**Figure 7:**
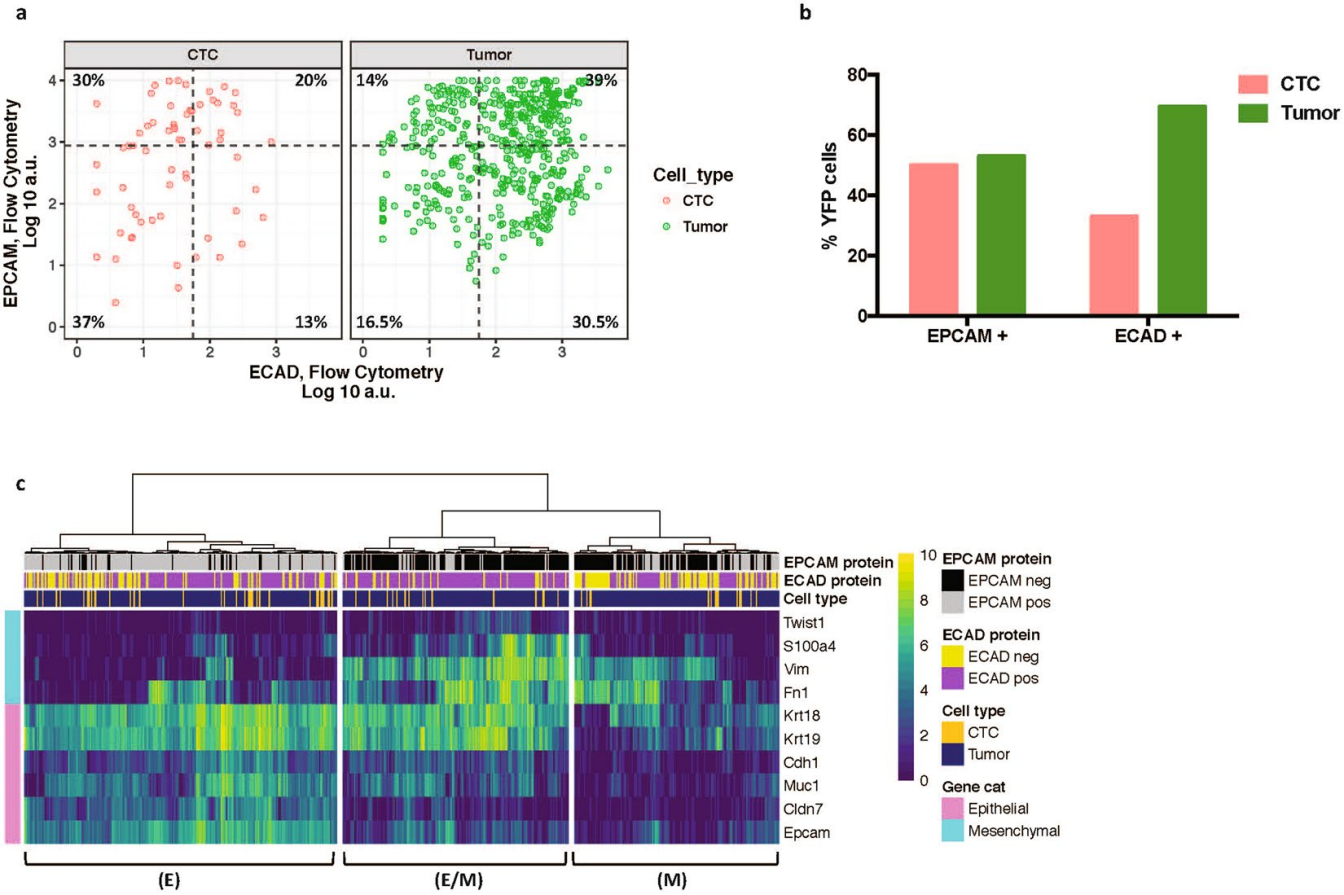
Single cell analysis of sorted CTCs and tumor cells from KPCY mice. (**a**) Comparison of EPCAM and ECAD expression by flow cytometry for CTCs and matched tumor cells. Gates are based on fluorescence minus one (FMO) controls. Percent of YFP+ CTCs and tumor cells expressing EPCAM or ECAD are quantified at right. (**b**) Expression of EMT genes in matched CTCs and tumor cells sorted from KPCY mice. (i) Epithelial (E) cluster (ii) Mesenchymal (M) cluster (iii) Hybrid (E/M) cluster. Scale bar refers to log 2 MI counts.

Flow cytometry analysis of sorted single cells allowed us to first compare surface expression of EPCAM and ECAD protein on matched CTCs, tumor cells, and WBCs. As expected, WBCs did not express detectable levels of either marker as measured by flow or RNA expression (Supplementary Fig. S5). We found that a much lower proportion of CTCs expressed ECAD at the cell surface (33%) as compared to tumor cells (69.5%), suggesting EMT may be associated with dissemination of tumor cells as has been reported previously in this model and others^13,49^. A similar proportion of CTCs and tumor cells expressed EPCAM, at 50% and 53%, respectively. 30% of CTCs and 14% of tumor cells maintained expression of EPCAM even upon loss of ECAD (Fig. 7a).

In order to more fully define the molecular features of CTCs and matched tumor cells, we also assessed the RNA expression of several known epithelial and mesenchymal genes. Index-sorting allowed us to link transcriptional profiles to EPCAM and ECAD protein expression. Unsupervised hierarchical clustering revealed three distinct transcriptional profiles, namely (i) an ‘epithelial’ subtype (E) expressing high levels o*f Epcam, Cdh1, Muc1* and cytokeratins, which was enriched for EPCAM protein+ cells, (ii) a ‘mesenchymal’ subtype (M) characterized by low expression of epithelial transcripts and concomitant increased expression of mesenchymal genes such as *Vim* and *Fn1*, and (iii) a ‘hybrid’ subtype (E/M) that has high expression of both mesenchymal and epithelial transcripts (Fig. 7b). This latter observation is consistent with previous reports from this preclinical model as well as in clinical samples^6,13,51^. These proof-of-principle experiments demonstrate that our workflow is capable of capturing rare cells and studying single cell heterogeneity from a complex biological sample in a robust manner.

## Discussion

Flow cytometry provides detailed, quantitative information about single cells and is routinely used in research and clinical settings^52–54^. One of the main limitations in using flow cytometry for analysis of rare cells in a complex matrix such as blood is that it is time-consuming and cumbersome to detect such cells among the billions of unwanted cells such as WBCs, RBCs, and platelets in a tube of blood. Thus, the ability of even a sophisticated cell sorter to efficiently isolate rare cells is severely compromised in cases of high cellularity. By integrating (i) immunomagnetic depletion of unwanted WBCs and RBCs, (ii) microfluidic acoustic washing and debris removal in-line with cell sorting to minimize cell loss and to achieve highest enrichment of CTCs, and (iii) an optimized nozzle configuration for simultaneous capture of single cells and clusters, we have developed a workflow that is a cost-effective and clinically-deployable approach for molecular analysis of enriched, sorted CTCs.

A significant advantage of this methodology is its versatility in terms of the biomarkers that can be assessed due to a large repertoire of commercially available antibodies and fluorescent probes. Other detection techniques such as RNA probes^55^ and molecular beacons that bind intracellular RNA transcripts^56^ as well as aptamers that can be generated to recognize virtually any cell surface marker^57,58^, can also be easily adapted for this approach. Further, cell sorters offer considerable flexibility in modes of collection including different sized tubes, multi-well plates, or even microscope slides depending on the downstream application. The sorted cells remain viable and could be used for generation of CTC-derived cell lines^59,60^ or CTC-derived xenografts^61^, which can serve as personalized models to screen for drug sensitivities or perform functional studies. Further, this workflow can be modified to isolate any relevant cell subset from peripheral blood, such as T regulatory and circulating fetal cells. It offers the flexibility to positively select any cell type of interest based on the markers used for phenotypic analysis. Or, the magnetic microparticles can be conjugated to a cocktail of antibodies or streptavidin in order to deplete any unwanted cells, and the remaining enriched cells can be sorted in an unbiased manner. This approach is relevant in the era of immunological therapies such as chimeric antigen receptor T-cell (CAR-T) therapy^62^ where (i) CTCs can be isolated from WBCs in peripheral blood using the workflow as described, and (ii) the magnetically depleted WBCs can be recovered and CAR-T cells can be further analyzed by depleting non-T cell populations.

A critical feature of our approach is the ability to perform high-throughput, high-dimensional and high-resolution (single-cell) molecular analysis including whole transcriptome analysis and the BD Precise™ Assays for RNA quantification. Although other platforms can efficiently isolate rare cells for single-cell molecular analysis, most of them require an additional, manual cell picking step that is highly labor intensive and not scalable for a clinical assay^37,51^. Our results suggest that highly purified cell populations can be isolated, and that the workflow is well suited for rapid adoption in a clinical laboratory setting where commercial flow cytometers are regularly used. Further, the gentle sample processing workflow minimizes cell loss and maintains the phenotypic and transcriptional profile of isolated cells. BD Precise assays can be easily customized to perform 3′ quantitative RNA sequencing using a targeted panel of up to 240 genes of interest in a cost-effective manner by pooling up to 1152 indexed single cells in one sequencing run. Our integrated approach is thus well-suited for performing molecular subtyping using CTCs, particularly in the setting of pancreatic and other cancers where tumor tissue biopsies are difficult to obtain^41^. Downstream analysis can also be extended to whole exome sequencing^63^, targeted DNA sequencing, or copy number analysis^64–66^ to assess tumor clonality or for mutational profiling. The index-sorting feature allows phenotypic signatures for a cell or cluster to be retained as gene expression or mutational signature is measured, which is unique to this platform. The ability of this technique to delineate heterogeneity at the single cell level is exemplified by our analysis of the epithelial and mesenchymal phenotypes of CTCs. Specifically, our characterization of rare CTCs from a preclinical model of pancreatic cancer suggest that neither EPCAM nor ECAD alone is sufficient to capture the whole spectrum of CTCs, and additional biomarkers are needed to enable sensitive CTC isolation. We identified three distinct CTC sub-populations based on single cell gene expression analysis, including a hybrid epithelial/mesenchymal population that could only be resolved on a single-cell level. Previous studies have shown that this hybrid phenotype correlates with increased plasticity and invasive potential^67^. Finally, an advantage of our system is the ability to recover viable cells for functional studies, which in combination with index sorting, can then be correlated to a novel phenotype with potential prognostic value.

In summary, our proof of principle study demonstrates that this workflow can be used to isolate and characterize purified rare circulating cells and clusters in peripheral blood. To our knowledge, this is the first pre-clinical demonstration of the feasibility of a platform that integrates rare cell enrichment with single cell and cluster isolation, and downstream analysis. Future studies will seek to adapt and, if necessary, re-optimize this workflow for human control and cancer patient samples, as well as expand to other cancers. Experiments will be conducted to increase sample throughput by increasing the fluidic path of the acoustic focusing chip, which can concentrate the sample or by adding multiple chips in parallel. We will also aim to improve cell recovery by experimenting with different types of magnetic tubing and by reducing the dead volume. Using single cell analysis of pre-clinical blood samples, we will also seek to discover CTC biomarkers that can be used to improve sensitivity of detection of human CTCs, many of which have been shown to lack expression of epithelial markers such as ECAD and EPCAM^68^. Finally, while our work was conducted using a single type of flow sorter, this pre-enrichment approach can be easily combined with other platforms, and future studies will be performed to demonstrate the compatibility of pre-enrichment with other commercially available flow sorters. In summary, this novel proof of concept study paves the way to real-time CTC analysis for biological insight into the metastatic process as well as pre-clinical and, eventually, clinical patient monitoring.

## Methods

### Fabrication of pre-enrichment platform

#### Magnetic depletion

The first step of the pre-enrichment platform consists of a high field high gradient magnet assembly enclosing magnetic separation tubing to remove blood cells that have been labeled with magnetic microparticles. The magnetic assembly is composed of Neodymium magnet (3 pieces of 2″ × 1/2″ × 1/2″ bar magnet, Applied Magnets, Plano, TX) and magnetic flux guide made of soft magnetic material (prototype machined and developed at BD Biosciences). The pole shaped magnetic flux guides are attached to the bar magnets and create a high field high gradient magnetic region that is 2 mm wide between the two opposing guides. The finite element method (FEM) calculated magnetic field and gradient of the region is 1.6 Tesla and 2.5 Tesla/m at 0.1 mm distance to the gap. A thin wall tubing (0.042″ inner diameter × 0.1082″ outer diameter × 6″ length, Tygon®, Cole Parmer, Vernon Hills, IL) that sits on the magnetic gap is used as a separation column. Cells bound with magnetic microparticles are pulled and held to the tubing wall when the sample passes through the magnetic column.

#### Acoustic focusing

The second step of the pre-enrichment consists of a microfluidic chip with an acoustic standing wave established in the channel that allows for separation of cells from debris including RBC lysate, platelets and unbound antibody conjugates. The microfluidic chip is made of glass with a wet etched channel (375 um wide × 125 um deep × 70 mm long, Micronit) in the middle and two inlets and two outlets at the ends. A 2 MHz piezoelectric transducer (lead zirconate titanate, EBL Products, Inc. East Hartford, CT) is attached to the glass chip to generate vibrational energy and the acoustic energy is resonant between the walls of the channel. This leads to the formation of an ultrasonic standing wave with energy node in the center of the channel. A laminar flow is established in the channel when sample enters from the inlets on either side and wash buffer enters in the center. When the acoustic field is applied, cells are forced to the pressure node in the central stream of the channel, but debris and original buffer remain at the side streams. Washed cells exit the chip from the central outlet while small debris and unbound reagent exit at the side outlets. The density of wash buffer creates another energy barrier to select cells from debris.

A pressure driven fluidic control system controls the flow rates in and out of the magnet column and acoustic chip, and sends the enriched tumor cells directly to the flow cell of the BD Influx™ cell sorter. Using a 200 μm nozzle on the cell sorter results in a sheath pressure of 3.5 psi, which generates a positive flow through the pre-enrichment system and sorter. All flow rates through the system are measured with flow meters (Sensirion, Switzerland). For all experiments in this report, flow to cell sorter was 60–70 μl/min, wash flow rate was 90–110 μl/min, waste flow rate was 130–140 μl/min, and sample consumption was about 100 μl/min.

### Cell Culture

Mouse cell lines PD7591, PD483 and PD798 had been previously generated from pancreatic tumor tissue isolated from Pdx1-cre, Kras^LSL-G12D^, p53^L/+^, Rosa^YFP/YFP^ (KPCY) mice^49^. They were cultured in Dulbecco’s Modified Essential Media supplemented with 10% Fetal Bovine Serum and 1x Penicillin-Streptomycin at 37 °C with 5% CO_2_.

### *In vitro* generation of cell clusters

*In vitro* clusters were generated as previous described^17^. Briefly, adherent cells (PD7591 and PD798) were trypsinized and cultured in a 6 cm suspension culture dish with rocking for 12–16 h in a 37 °C incubator at 5% CO_2_ at a density of approximately 1 million cells per ml in 5 ml media. 100 μl of cell suspension was then spiked into 0.5 ml of mouse blood and subjected to the sample preparation workflow described above. 1000 cells (events) were sorted into 4 wells of a 12-well plate for each sample. 5–6 individual images were collected per well and the counts were added to obtain a representative count for each well. The mean for 4 wells was reported. In order to assess the distribution of clusters prior to sorting, 1 ml of the cell suspension prior to spiking into blood was imaged in a 6 cm dish. 7 representative images were collected and size and number of clusters were quantified. All wells were stained with Hoechst 33342 and imaged on an Olympus IX71 inverted multicolor fluorescent microscope.

### Mouse blood and tissue collection

All mouse work was performed in compliance with the National Institutes of Health guidelines for animal research and as per protocols approved by the University of Pennsylvania Institutional Animal Care and Use Committee. Blood was obtained from healthy and tumor-bearing mice by cardiac puncture and collected in either heparin or EDTA BD Vacutainer® blood collection tubes. Matched tumor tissue was also collected at necropsy.

### Sample preparation

Adherent cells were dissociated using 0.25% Trypsin-EDTA, resuspended in media and spiked into 1 ml of healthy mouse blood. The sample was stained with CD45-APC (Clone 30-F11) (BD Biosciences, San Jose, CA), EPCAM-BV711 (Clone G8.8) (BD Biosciences, San Jose, CA) and ECAD-PE (Clone DECMA-1 (Biolegend, San Diego, CA)) at a 1:100 dilution for 20 minutes at room temperature with shaking. This was followed by incubation with BD IMAG™ anti-mouse Ter-119 and anti-APC magnetic particles at 1:10 dilution for 20 minutes at room temperature with rocking. Then, 4x volume of RBC Lysis Buffer (G-Biosciences, St. Louis, MO) was added to the unwashed sample and it was incubated for 15 minutes with shaking. Prior to analysis on the flow cytometer, DAPI (4′,6-Diamidino-2-Phenylindone) was added at a final concentration of 1 μg/ml. The same procedure was followed for blood obtained from tumor-bearing mice. For tumor samples, tumor tissue was cut into smaller pieces with scissors, followed by incubation with 2 mg/ml Collagenase IV at 37 °C for 20 minutes with intermittent vortexing. The suspension was then passed through a 70 μM cell strainer, washed with PBS and stained with antibodies against CD45, EPCAM and ECAD (1:100 dilution) for 20 minutes at 4 °C. Samples were washed with PBS + 5% fetal bovine serum and DAPI at 1 μg/ml was added prior to sorting.

### Fluorescence-activated cell sorting

All cell sorting was performed on the BD Influx™ cell sorter using BD FACS™ Sortware software. The standard plate holder apparatus for the Influx was filed down to accommodate a 96-well cooling block (BioCision CoolRack®, San Rafael, CA), which could hold BD Precise™ Single Cell Encoding 96-well plates in a rigid position to enable sorting.

For WTA analysis, YFP positive cells were sorted into chilled 0.2 ml PCR tubes (Applied Biosystems, Foster City, CA) containing 5 μl of PBS (w/o Ca/Mg) and SUPERaseIn™ RNase inhibitor (Invitrogen, Carlsbad, CA) at 1 U/ml. The tubes were centrifuged at 300 *g* for 10 minutes followed by addition of 5 μl of 2x Single-Cell Lysis Buffer (Takara Bio USA, Inc., Mountain View, CA) with SUPERaseIn™ RNase inhibitor at 2 U/ml. The lysates were stored at −80 °C until cDNA synthesis.

For Precise workflow, cells were sorted directly into chilled BD Precise™ Single Cell Encoding 96-well plates containing lysis buffer, indexing dT primers and dNTPs. After sorting, plates were sealed with a foil cover, vortexed for 5–10 seconds, briefly centrifuged and frozen at −80 °C until further analysis.

### Whole transcriptome RNA sequencing

#### Library prep and sequencing

Whole lysate from sorted cells was used as the starting material for synthesis of cDNA using the Clontech SMART-Seq® v4 Ultra® Low Input RNA Kit for Sequencing (Takara Bio USA, Inc., Mountain View, CA). cDNA was fragmented to an average size of 300 bp using the Covaris E220 ultrasonicator. Sequencing libraries were prepared using the Low Input Library Prep Kit HT (Takara Bio USA, Inc., Mountain View, CA) and checked for quality using the Tapestation 2200 (Agilent, Santa Clara, CA) and Qubit dsDNA HS assay (Thermo Fisher Scientific, Waltham, MA). Samples were then sequenced on the Illumina NextSeq® 500 with paired-end 75 bp reads and a minimum depth of 3 million reads per sample.

#### Single cell RNAseq Alignment and Processing

FASTQ reads from RNA sequencing were aligned to mm10 reference genome using STAR aligner (version 2.5.2a)^69^ and gene-level counts were binned using featureCounts (version 1.4.6-p4)^68,70^. ERCC92 fasta sequence and gene feature annotation files were obtained from Thermo Fisher and combined with mm10 reference information. Gene level counts were upper-quartile normalized using the R package EDASeq and converted to transcripts per million (TPM) using the gene effective length^71^.

### BD Precise™ Analysis

Precise plates were prepared for sequencing following manufacturer’s instructions for the BD Precise™ Reagents kit. Briefly, cDNA was synthesized, samples containing well-specific indices were pooled, and gene targets were amplified using 20 cycles and a custom primer panel. Plate indexes were added during the library amplification stage and plate pools were quantitated using the KAPA Universal Illumina Library Quantification Kit (Roche, Pleasanton, CA) and the Agilent Bioanalyzer High Sensitivity DNA kit. 2 nM dilutions from each plate were pooled, 1.44 pM libraries were loaded onto the Illumina NextSeq. 500 with 30% PhiX, and 2 × 75 bp reads were sequenced. Reads were mapped, assigned to Molecular Indexes (MI), and corrected using the Bowtie v2 aligner-based BD Precise™ Targeted Analysis Pipeline. (MI) counts were subjected to quality checks before downstream analysis. Wells with less than 50 total MI were rejected as poor quality cells. Cells that did not express at least a total of 4 MI counts from housekeeping genes from our custom panel (*Reep5, Gapdh, Psmb2, Psmb4 and Vcp*) were also considered to be compromised. Any features that did not have at least 3 nonzero values and were not expressed in at least 10 cells were removed from analysis.

### Data analysis

Flow cytometric data analysis was performed using FlowJo and FCS Express software.

Data analysis including calculating cell recovery, effect of sample processing, assessment of cluster distribution, primary component analysis (PCA) and t-distributed stochastic neighbor embedding (t-SNE) analysis was performed using R statistical software^72^ and the Rtsne package^73–75^. Where logged RNA counts are shown, these values include a pseudocount of +1. Hierarchical clustering was also used to categorize genes with distinct behaviors between different cell types.

### Data availability

The datasets generated and analyzed during the current study are available in the GEOarchive repository GSE108287. These datasets can also be provided upon request.

## Electronic supplementary material

Supplementary Information
