# Supplementary material for: An integrated flow cytometry-based platform for isolation and molecular characterization of circulating tumor single cells and clusters
Source: Sci Rep. 2018 Mar 22;8:5035. doi: 10.1038/s41598-018-23217-5 (PMC5864750; doi:10.1038/s41598-018-23217-5)
Supplement: Supplementary file 1 — Supplementary Information [file 41598_2018_23217_MOESM1_ESM.pdf]

An integrated flow-cytometry based platform for isolation and molecular characterization of circulating tumor single cells and clusters

Neha Bhagwat[1], Keely Dulmage[2], Charles H. Pletcher, Jr [3], Ling Wang[2], William DeMuth[3], Moen Sen[4], David Balli[1], Stephanie S. Yee[4], Silin Sa[5], Frances Tong[2], Liping Yu[5], Jonni S. Moore[3], Ben Z. Stanger[1], Eric P. Dixon[2], Erica L. Carpenter[4]

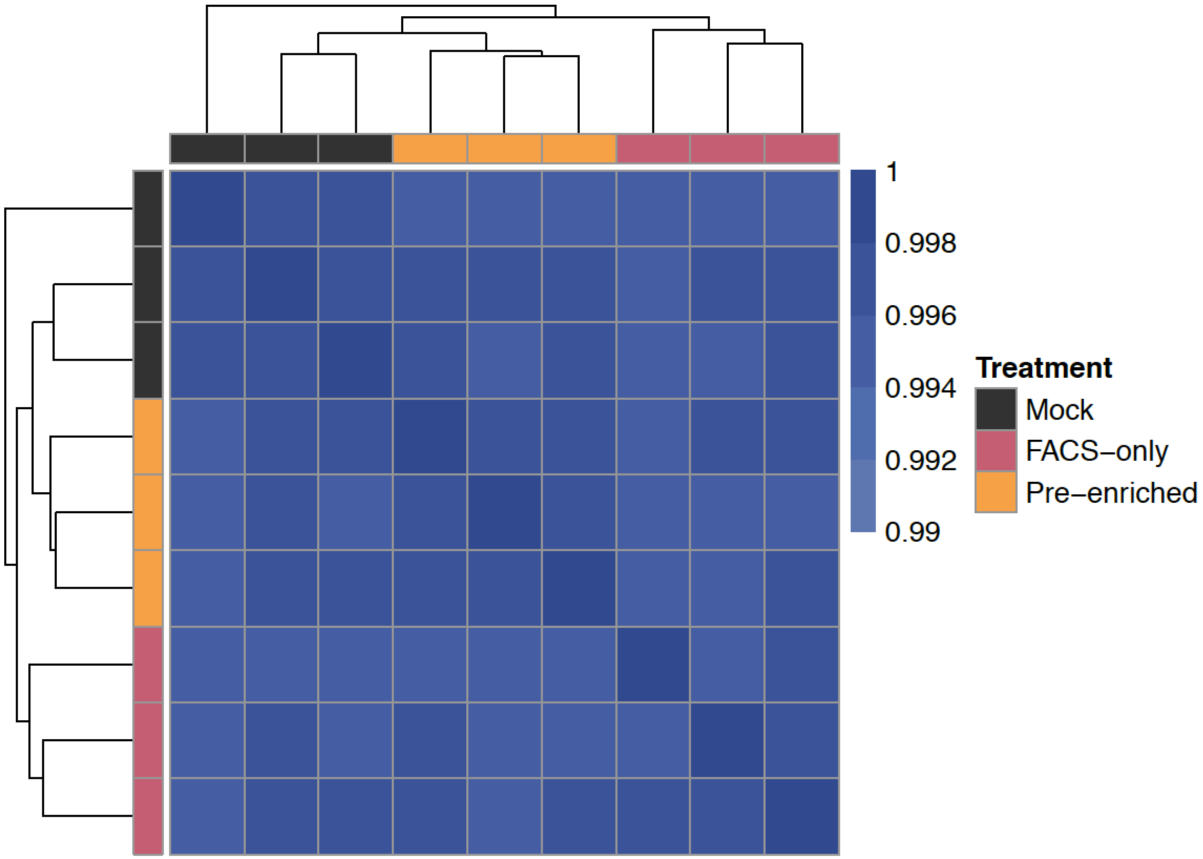

Supplementary Figure S1: Correlation matrix comparing gene expression profiles of PD483 cells as determined by whole transcriptome RNA sequencing. Cells were sorted through the pre-enrichment workflow (Pre-enriched), sorted through Influx only (FACS-only), or were incubated in PBS only on the bench (Mock). Scale refers to Pearson correlation coefficient. (n=3). 13,274 genes were considered in the analysis.

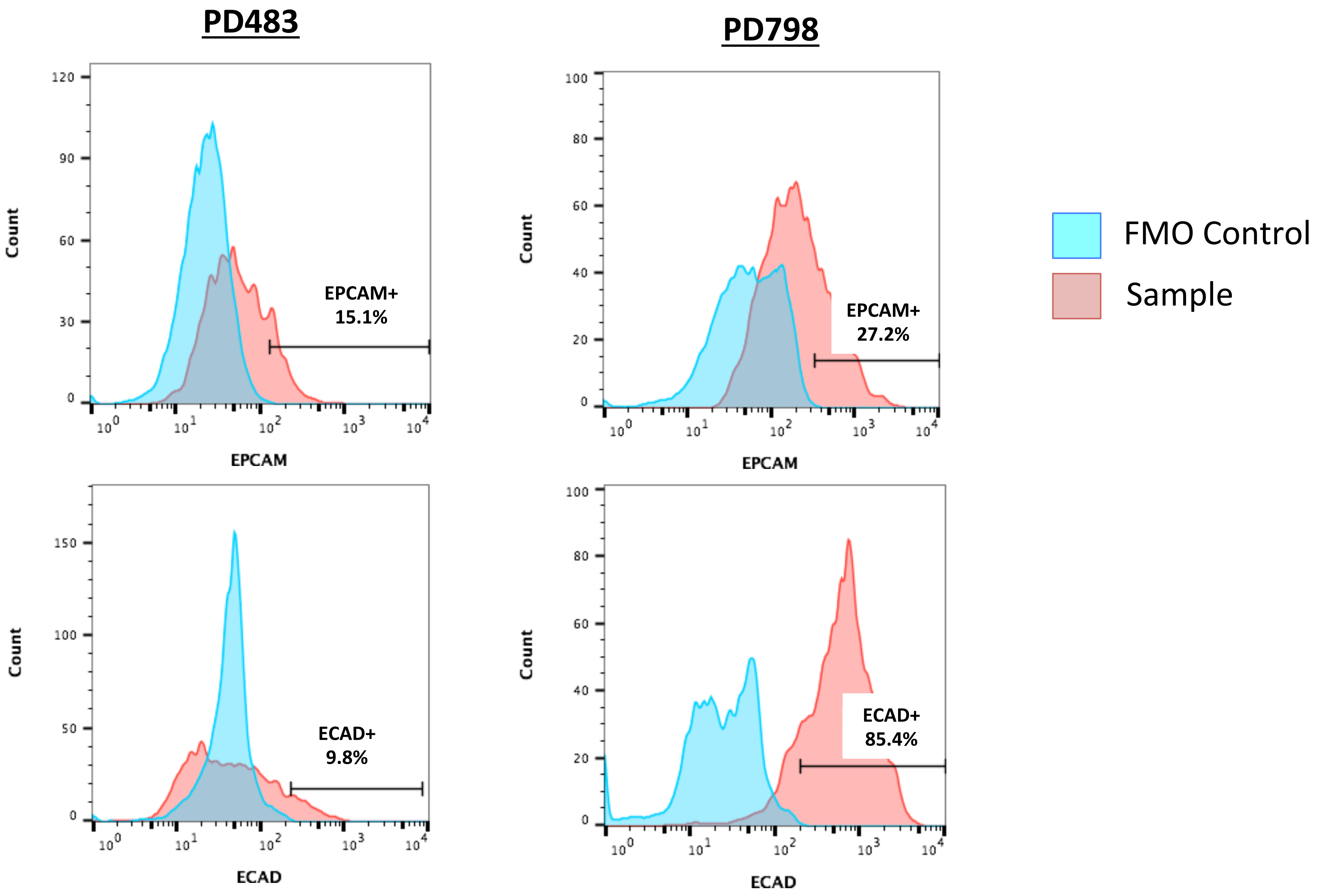

Supplementary Figure S2: EPCAM and ECAD expression in PD798 and PD483 by flow cytometry. FMO: Fluorescence minus one

a

% Uniquely Aligned Reads			
**Cell number**	**Min**	**Max**	**Mean**
**1**	80.51	88.95	86.56
**10**	84.67	87.81	86.65
**100**	84.81	88.18	86.57
**500**	85.07	86.29	85.75
**RNA**	87.77	87.98	87.88

b

# Uniquely Aligned Reads			
**Cell number**	**Min**	**Max**	**Mean**
**1**	5.22E+06	4.19E+07	1.26E+07
**10**	4.05E+06	3.14E+07	1.55E+07
**100**	3.82E+06	9.74E+06	5.83E+06
**500**	4.56E+06	9.01E+06	6.13E+06
**RNA**	8.33E+06	1.14E+07	9.88E+06

Supplementary Figure S3: Sequencing metrics from single and pooled cell sequencing of PD798. (a) Percent of reads, and (b) Total number of reads that uniquely aligned to the mouse reference genome.

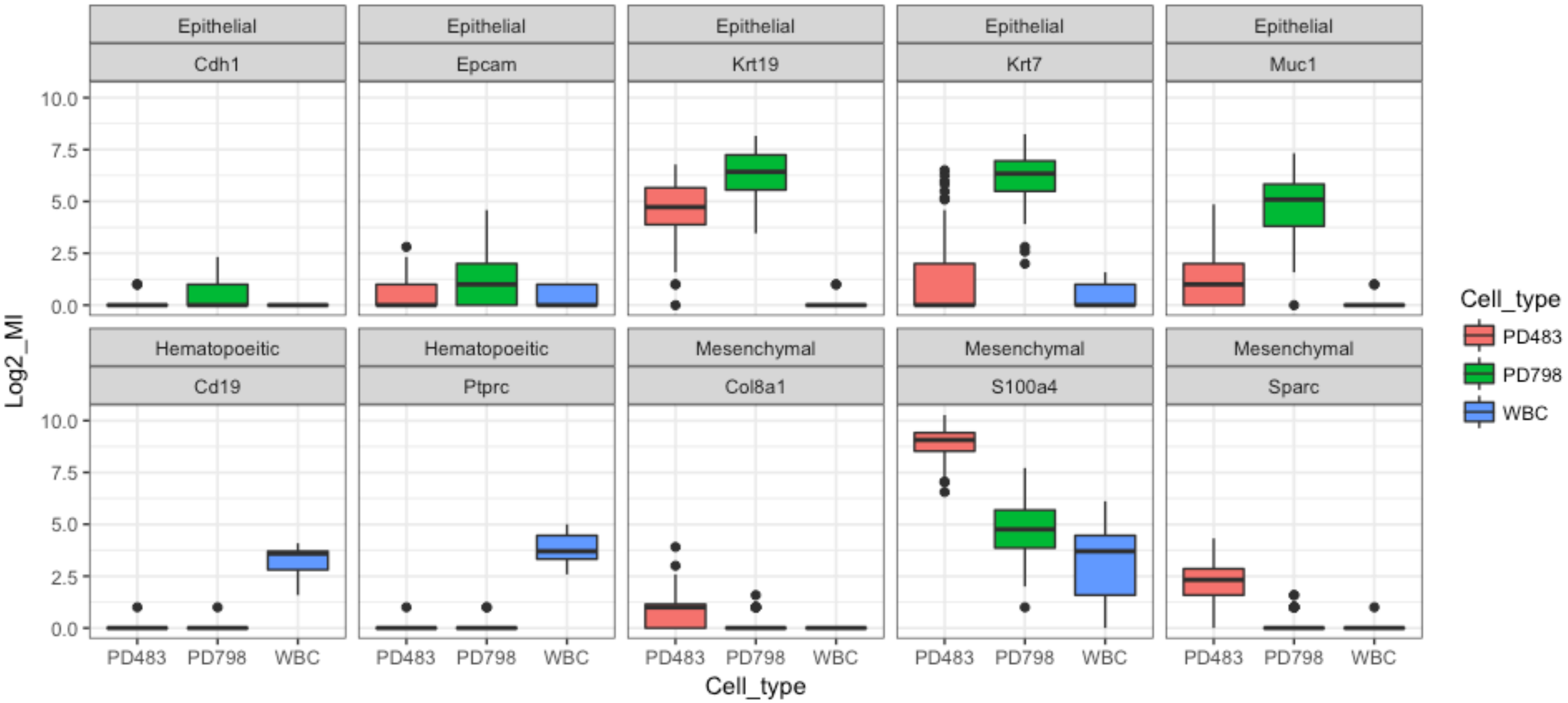

Supplementary Figure S4: Expression of select epithelial, mesenchymal, and hematopoietic genes in PD483, PD798, and WBCs using BD Precise™ assays.

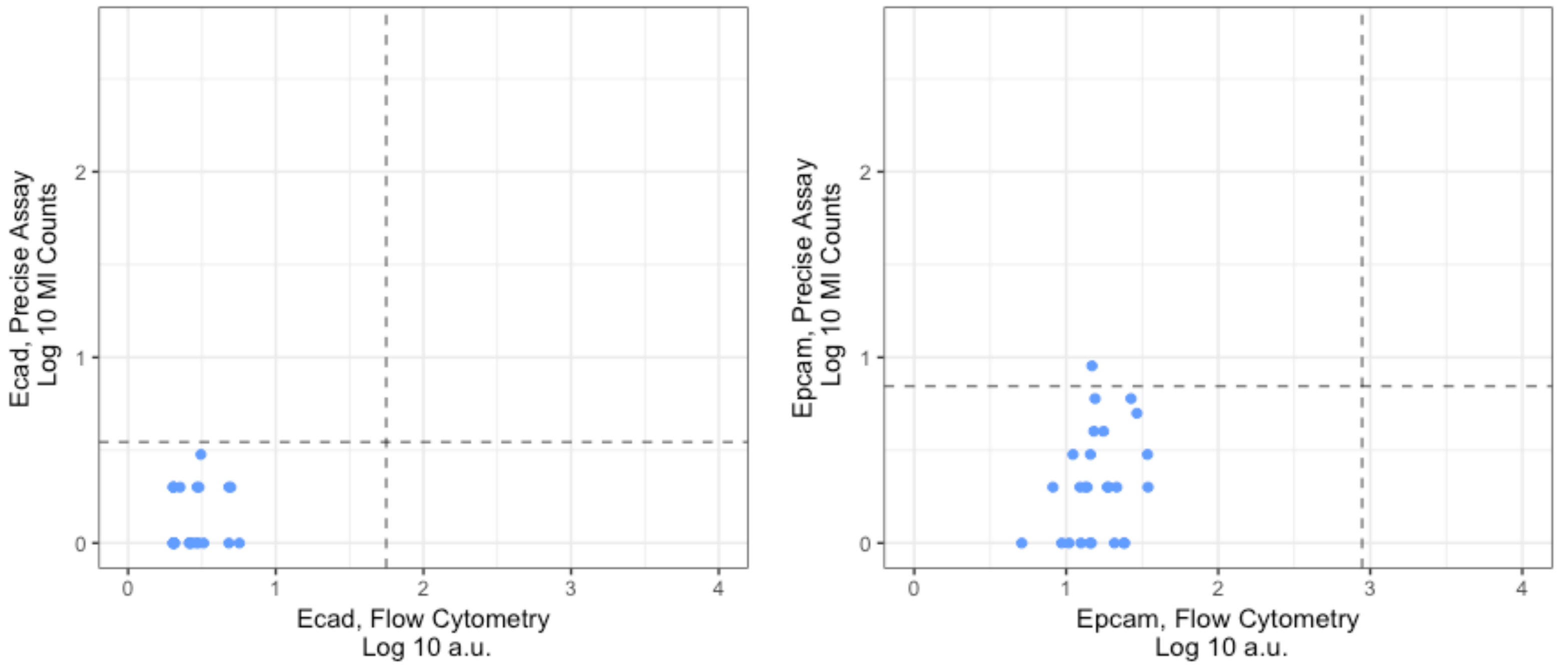

Supplementary Figure S5: Expression of EPCAM and ECAD in WBCs by flow cytometry and by Precise RNA quantification.

Supplementary Table S1: List of genes in BD Precise EMT panel

Apc	Col8a1	Hpse	Mmp9	Set	Zeb1
Brca1	Cst7	Hras	Msh2	Smad2	Zfp957
Brms1	Ctbp1	Htatip2	Mta1	Smad4	
Bub1	Ctnna1	Igf1	Mtss1	Snai1	
Ccl7	Ctsk	Il18	Muc1	Snai2	
Ccnb1	Ctsl	Il1b	Mycl	Sncg	
Ccne2	Cxcl12	Itga2b	Myc	Sparc	
Cd14	Cxcr4	Itga7	Ncam1	Src	
Cd19	Ddr2	Itgb3	Nf2	Sstr2	
Cd3e	Denr	Kiss1r	Nme1	Syk	
Cd44	Emc7	Kiss1	Nme4	Tcf20	
Cd82	Epcam	Kras	Nr4a3	Tgfb1	
Cdc20	Ephb2	Krt18	Palld	Tgfbr2	
Cdh11	Esrp1	Krt19	Pdgfrb	Tiam1	
Cdh1	Etv4	Krt20	Pdpn	Timp2	
Cdh2	Ewsr1	Krt7	Plaur	Timp3	
Cdh6	Fap	Krt8	Plk1	Timp4	
Cdkn2b	Fat1	Lum	Pnn	Tnc	
Chd4	Fcgr4	Mcam	Postn	Tnfsf10	
Chmp2a	Fgfr4	Mcm6	Psmb2	Tpbg	
Cldn2	Flt4	Mdm2	Psmb4	Trp53	
Cldn4	Fn1	Metap2	Pten	Trpm1	
Cldn7	Fxyd5	Met	Ptprc	Tshr	
Col12a1	Gapdh	Mgat5	Rab7	Twist1	
Col1a1	Gli1	Mmp10	Rb1	Vcp	
Col1a2	Gnrh1	Mmp11	Reep5	Vegfa	
Col3a1	Gpi1	Mmp13	Rorb	Vegfc	
Col4a2	Gypa	Mmp2	Rrm2	Vim	
Col5a1	Hgf	Mmp3	S100a4	Vps29	
Col6a1	Hprt	Mmp7	Serpine1	Wisp1	
